# Effects of Sugammadex on Post-Operative Pulmonary Complications in Laparoscopic Gastrectomy: A Retrospective Cohort Study

**DOI:** 10.3390/jcm9041232

**Published:** 2020-04-24

**Authors:** Jiwon Han, Jung-Hee Ryu, Bon-Wook Koo, Sun Woo Nam, Sang-Il Cho, Ah-Young Oh

**Affiliations:** 1 Department of Anesthesiology and Pain Medicine, Seoul National University Bundang Hospital, 82, Gumi-ro, Bundang-gu, Seongnam-si, Gyeonggi-do 13620, Korea; hanjiwon@snubh.org (J.H.); jinaryu74@gmail.com (J.-H.R.); tendong2@gmail.com (B.-W.K.); nsw116@snubh.org (S.W.N.); dapack@naver.com (S.-I.C.); 2 Department of Anesthesiology and Pain Medicine, Seoul National University College of Medicine, 103, Daehak-ro, Jongno-gu, Seoul 03080, Korea

**Keywords:** laparoscopic gastrectomy, neuromuscular blocking agent, post-operative pulmonary complications, sugammadex

## Abstract

The use of sugammadex can reduce post-operative residual neuromuscular blockade, which is known to increase the risk of post-operative respiratory events. However, its effect on post-operative pulmonary complications is not obvious. This study was performed to evaluate the effects of sugammadex on post-operative pulmonary complications in patients undergoing laparoscopic gastrectomy between 2013 and 2017. We performed propensity score matching to correct for selection bias. Post-operative pulmonary complications (i.e., pneumonia, respiratory failure, pleural effusion, atelectasis, pneumothorax, and aspiration pneumonitis) were evaluated from the radiological and laboratory findings. We also evaluated admission to the intensive care unit after surgery, re-admission or an emergency room visit within 30 days after discharge, length of hospital stay, re-operation, and mortality within 90 days post-operatively as secondary outcomes. In the initial cohort of 3802 patients, 541 patients were excluded, and 1232 patients were analyzed after propensity score matching. In the matched cohort, pleural effusion was significantly reduced in the sugammadex group compared to the neostigmine group (neostigmine 23.4% vs. sugammadex 18%, *p* = 0.02). Other pulmonary complications and secondary outcomes were not significantly different between the groups. In comparison to neostigmine, the use of sugammadex was associated with a lower incidence of post-operative pleural effusion in laparoscopic gastrectomy.

## 1. Introduction

In general anesthesia, neuromuscular blockade provides appropriate surgical conditions and patient safety by inhibiting involuntary movement of the patient [1,2]. On the other hand, it also increases the risk of immediate post-operative critical respiratory events, such as hypoxemia and upper airway obstruction, mainly due to residual neuromuscular blockade [3,4]. Reversal agents are commonly used to reduce residual neuromuscular blockade. Traditionally, anticholinesterases such as neostigmine are used for reversal, but these agents have some limitations. Neostigmine increases the acetylcholine in both the nicotinic and muscarinic receptors, so cholinergic side effects (e.g., bradycardia, bronchoconstriction, post-operative nausea and vomiting) can occur. To prevent such side effects, choline antagonists, such as glycopyrrolate or atropine, should also be administered; these can lead to a dry mouth, tachycardia, and urinary retention. In addition, the reversal of a deep neuromuscular blockade by neostigmine is impossible. Neuromuscular reversal guidelines recommend administering neostigmine when a train of four (TOF) count of at least two is confirmed [5]. In addition, neostigmine overdose is known to cause a paradoxical neuromuscular block [6,7]. Sugammadex forms a complex with aminosteroidal agents to induce the rapid and complete reversal of even deeper neuromuscular blockade, and it significantly reduces post-operative residual blockade [8,9,10]. Sugammadex enables deep neuromuscular blockade, resulting in an improved surgical condition score and improved surgeon satisfaction, especially in laparoscopic surgery [11,12]. In addition, sugammadex does not have cholinergic side effects. Despite these many advantages, the effects of sugammadex on post-operative patient outcomes (e.g., mortality, morbidity, and complications) are controversial [13,14,15]. This study was performed to investigate the relationships between post-operative pulmonary complications and types of reversal agent (sugammadex vs. neostigmine) in laparoscopic gastrectomy. The secondary purpose was to evaluate the relationships between types of reversal agent and other post-operative outcomes, including re-operation within 90 days, intensive care unit (ICU) admission, re-admission or an emergency room visit within 30 days, length of hospital stay, and mortality within 90 days.

## 2. Materials and Methods

This retrospective observational study was conducted after receiving approval from the Institutional Review Board of Seoul National University Bundang Hospital (approval number: B-1801-447-004); the requirement for informed consent was waived due to the study’s retrospective nature.

Data from the 3802 patients receiving laparoscopic gastrectomy under general anesthesia at Seoul National University Bundang Hospital between January 2013 and December 2017 were analyzed retrospectively. We excluded patients under 20 years old, those with conversion to laparotomy, and those with other surgeries. In addition, we excluded cases in which succinylcholine or cisatracurium was used, both sugammadex and neostigmine were used, or neither was used.

The data were extracted from electronic medical records, including demographic data, anesthetic records, laboratory findings, and reviews of chest radiography and chest computed tomography reports by radiologists blinded to the reversal group. All surgical patients underwent their first chest radiography on day 1 or 2 after surgery. Follow-up chest radiography or computed tomography was performed in patients with abnormalities on the first radiograph or in those with symptoms such as fever, coughing and sputum. We reviewed the radiological results up to 7 days after surgery.

Sugammadex 2 or 4 mg/kg or neostigmine 20–50 µg/kg with 0.4 mg glycopyrrolate was used for the reversal of rocuronium. The neuromuscular blockade status was monitored before administration of the reversal agents to determine the correct doses. Both quantitative and qualitative monitoring were allowed for this purpose, but monitoring until full recovery was not mandatory and the possibility of residual- neuromuscular blockade could not be ruled out.

Fentanyl-based patient-controlled analgesia was applied to all the surgical patients for post-operative pain management. Fentanyl (50 µg intravenously) was most commonly used as a rescue analgesic. A transdermal fentanyl patch (50 µg/h), 10 mg nalbuphine, 25 mg pethidine, or 100 mg tramadol was used in patients requiring additional analgesics.

The primary outcomes were pulmonary complications within 7 days post-operatively defined according to European perioperative clinical outcome (EPCO) guidelines [16]. Respiratory infection was diagnosed based on chest radiography and chest computed tomography results, and at least one of the following: white blood cell count ≥ 12,000/mm^3^ or body temperature ≥ 38 ℃ within 7 days post-operatively. Respiratory failure was defined as PaO_2_ < 60 mmHg or SpO_2_ < 90%. Pleural effusion, atelectasis, pneumothorax, and aspiration pneumonitis were determined based on the radiological findings (Table 1).

The secondary outcome was re-operation within 90 days post-operatively, admission to the ICU after the operation, re-admission or an emergency room visit within 30 days after discharge, length of hospital stay, and mortality within 90 days post-operatively.

### Statistical Analysis

Baseline cohort’s characteristics were compiled as the mean and standard deviation for numerical variables, numbers and percentages for categorical variables. The student *t*-test and the χ^2^ test were used for comparing the two groups. The administration of sugammadex or neostigmine was not randomly assigned, and in order to reduce selection bias in non-randomized treatment, a propensity score matching (PSM) was applied. Propensity score means the probability of being assigned to a treatment group, estimated by the given covariates. In observational study, PSM can be used to balance the covariates between non-randomized groups.

Possible variables that could affect post-operative pulmonary complications were included as follows: patient characteristics, American Society of Anesthesiology (ASA) class; anemia defined as a pre-operative hemoglobin level < 12 g/dL for women and <13 g/dL for men; glomerular filtration rate; pre-operative comorbidity; smoking history; pre-operative lung disease; pulmonary function test; type of surgery and diagnosis; anesthetic agents; anesthetic time; application of positive end expiratory pressure during surgery; peak inspiratory pressure; intraoperative infusion of crystalloid and colloid; transfusion; urine output; estimated blood loss, and infusion of inotropics and vasopressors.

The covariates were matched at a 1:1 ratio with a 0.15 caliper. After PSM, a cohort of 1232 matched patients was derived from an initial cohort of 3802 patients. The standardized mean difference (SMD) was used to confirm the balance between the two groups; an SMD < 0.1 indicated an appropriate balance between the two groups. The matched patient characteristics and outcomes were analyzed by the chi-square test, or *t*-test, as appropriate. In all the analyses, *p* < 0.05 was taken to indicate statistical significance. PSM were performed by R program (version 3.5.2; www.r-project.org), while the chi-square and *t*-test were performed by SPSS software (version 25.0; IBM corp, Armonk, NY, USA).

## 3. Results

**Figure 1:**
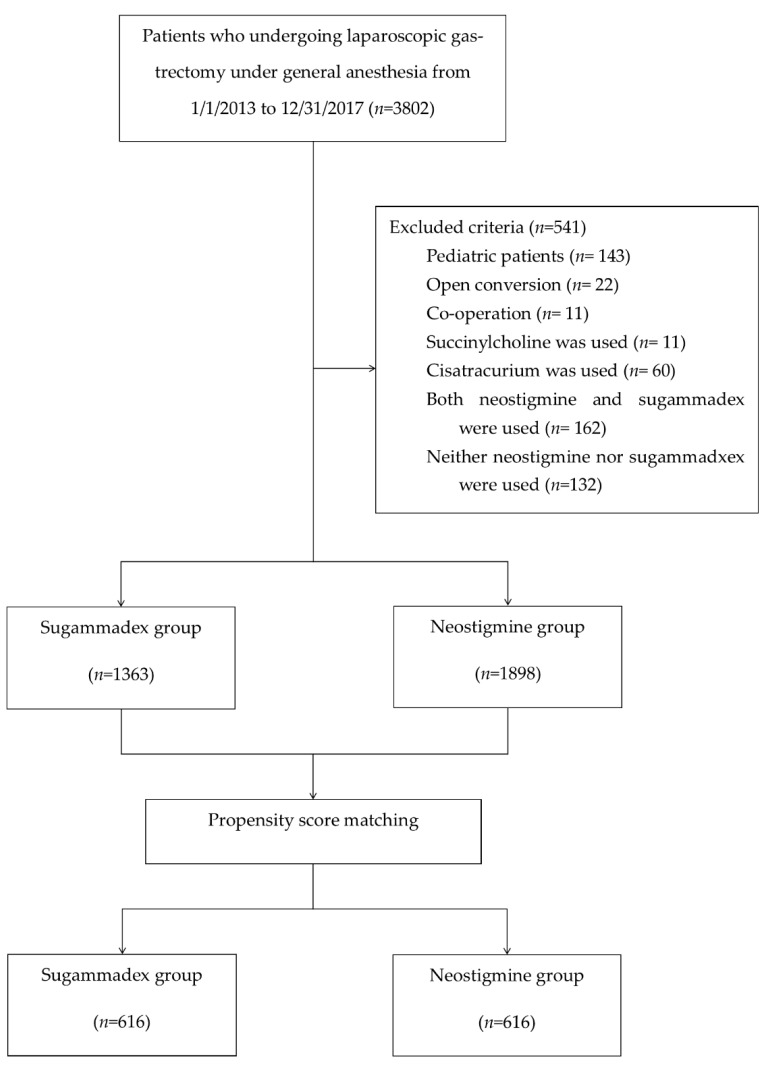
Flow chart of the study population.

In the initial cohort of 3802 patients who underwent laparoscopic gastrectomy between January 2013 and December 2017 at Seoul National University Bundang Hospital, 541 patients were excluded. However, the 1363 patients who received sugammadex, and the 1898 patients who received neostigmine were included in the analysis (Figure 1). 

Because these patients were not randomly assigned, there were statistically significant differences (*p* < 0.05) between the sugammadex group and the neostigmine group across several variables, including type of operation; anesthetic agent; application of positive end expiratory pressure; intraoperative colloid infusion amount; estimated blood loss; urine output; intraoperative use of ephedrine, phenylephrine, norepinephrine, atropine, and esmolol. PSM were performed for all the measured variables. After matching, 1232 patients consisting of 616 per group were finally analyzed. The patients’ characteristics and SMD values for the matched cohort are listed in Table 2; all SMD values were <0.1, indicating that a balance was achieved between the groups. As expected, following PSM, there were no significant differences between the groups in any of the measured variables.

The outcomes for the matched cohort are shown in Table 3. There was a statistically significant difference in the pleural effusion rate: 18% in the sugammadex group vs. 23.4% in the neostigmine group (*p* = 0.02). These patients received 3–5 L/min oxygen according to the surgical treatment policy, but no patient developed further symptoms or signs of infection, or required invasive treatment, such as thoracentesis. No statistically significant differences were observed between the groups in terms of overall and other pulmonary complications, and the groups did not differ significantly in terms of secondary outcomes, such as re-operation within 90 days post-operatively, admission to the ICU after the operation, re-admission or an emergency room visit within 30 days after discharge, length of hospital stay, and mortality within 90 days post-operatively (Table 4).

## 4. Discussion

This single-centre retrospective observational study revealed that the post-operative pleural effusion rate was lower in the sugammadex group compared with the neostigmine group. However, the overall incidence of other pulmonary complications, including respiratory infection, respiratory failure, atelectasis, pneumothorax, and aspiration pneumonitis, did not differ significantly between the groups. Secondary outcomes, including re-operation within 90 days, post-operative ICU care, re-admission or an emergency room visit within 30 days, length of hospital stay, and mortality within 90 days, did not differ significantly between the two groups.

Stomach cancer is the second most common cause of cancer-related deaths and is the fourth most common malignancy worldwide [17]. Laparoscopic gastrectomy was found to be more effective than open gastrectomy in reducing intraoperative blood loss, post-operative complications, and reducing hospital stays [18,19]. However, the incidence of pulmonary complications did not differ from that of open gastrectomy [20]. In particular, upper abdominal surgery is a risk factor for post-operative pulmonary complications, and a systematic review reported an odds ratio of 2.91 (95% Confidence Interval: 2.35–3.60) [21].

Pleural effusion may occur due to an imbalance between hydrostatic pressure and osmotic pressure in lung capillaries and interstitium. As residual neuromuscular blockade inhibits respiratory muscular function and lung expansion, the negative pressure in the pleural cavity may be reduced, which could lead to pleural effusion [22,23]. A possible explanation for our results is that post-operative residual neuromuscular blockade is reduced by sugammadex compared to neostigmine; hence pleural effusion was also reduced. Post-operative pleural effusion is common after upper abdominal surgery, and is considered benign and not mandating further intervention if there are no symptoms or signs of infection, because most cases resolve spontaneously within a few days [24]. However, caution is needed to prevent hypoxemia or further development to more serious complications, such as atelectasis or pneumonia.

A systematic review showed that sugammadex reversed neuromuscular blockade faster than neostigmine and decreased post-operative residual blockade. In addition, there have been several studies regarding how sugammadex affects various outcomes of patients. Sugammadex was shown to reduce post-operative nausea and vomiting because of the rapid recovery of muscle strength and the absence of the cholinergic side effects of neostigmine [25]. Some studies showed that sugammadex extended coagulation profiles and affected surgical bleeding, but these observations remain controversial [26,27]. A recent study showed that sugammadex was related to a lower incidence of re-admission, shorter hospital stay, and reduced hospital costs [28]. In addition, post-operative pulmonary complications have been studied. In sleeve gastrectomy, post-operative SpO_2_ was improved, but there were no differences in respiratory events such as desaturation requiring management, reintubation, and ICU admission [8]. A retrospective study showed that reversal with sugammadex was associated with a reduced risk of pulmonary outcomes in elderly patients of ASA class 3 or 4. The authors suggested that reversal with sugammadex would be beneficial in elderly patients [29]. On the other hand, the POPULAR multicentre, prospective observational cohort study showed no difference in the pulmonary complication rate between sugammadex and neostigmine use [30]. However, experts’ opinions that followed pointed out that the study was based on inappropriate use of neuromuscular blocking agents or reversal agents, based on the facts that only 40% of the studied patients were objectively monitored, the portion was even lower, and only 16.5% of patients had a documented TOF ratio of at least 0.9 at the time of extubation [31,32].

The reported incidence of post-operative pulmonary complications ranges from 5% to 90%, indicating a wide range depending on the definitions or criteria of pulmonary complications, patient populations, and types of surgery [33,34]. The PERISCOPE study showed an incidence of pulmonary complications, according to EPCO definitions, of 21.4% in upper abdominal operations [35]. A previous study indicated an incidence of 6.8% for pulmonary complications of laparoscopic gastrectomy, of which pleural effusion was reported as 2.16%, compared to 18%–23.4% in our study. This discrepancy may have been due to the difference in the definition of pleural effusion as a chest radiological examination requiring percutaneous intervention [36]. In the present study, the total pulmonary complication rate was 47.9%, which is approximately the median value of the published rate and somewhat higher than in other studies because asymptomatic radiological abnormalities were also detected (all patients underwent a post-operative chest radiological examination on the first or second day after surgery).

This study has some limitations. First, the patients reversed with sugammadex may have had a stronger intraoperative deep neuromuscular blockade compared to those administered neostigmine. Indeed, previous studies have revealed that intraoperative deep neuromuscular blockade during bariatric surgery is related to a reduced incidence of postoperative surgical complications [37,38]. However, its effect on pulmonary complications is not clear. Second, the retrospective observational design may have failed to extract information on possible confounding factors, such as intraoperative ventilation strategies. We included positive end expiratory pressure and peak inspiratory pressure as confounding factors but did not include tidal volume, driving pressure, or recruitment maneuvers [39]. Third, only the pleural effusion was significantly lower in the sugammadex group, and it is difficult to represent overall pulmonary complications after surgery. Fourth, large amounts of data were lost through the PSM procedure.

Further research is needed to clarify the relationships between post-operative outcomes according to the use of sugammadex compared with neostigmine.

## 5. Conclusions

In conclusion, this single-centre retrospective observational study shows that the use of sugammadex as a reversal agent compared to neostigmine decreases the incidence of post-operative pleural effusion in patients undergoing laparoscopic gastrectomy. However, the overall incidence of pulmonary complications, including respiratory infection, respiratory failure, pleural effusion, atelectasis, pneumothorax, and aspiration pneumonitis, does not differ significantly between the groups. Further research is needed to clarify the clinical significance of post-operative pleural effusion.

## Figures and Tables

**Table 1:** Definition of post-operative pulmonary complications according to European perioperative clinical outcome (EPCO) guidelines.

Complication	Definition
Respiratory infection	Patient has received antibiotics for a suspected respiratory infection and met one or more of the following criteria: new or changed sputum, new or changed lung opacities, fever, white blood cell count > 12 × 10^9^ /L
Respiratory failure	Post-operative PaO_2_ < 8 kPa (60 mmHg) on room air, a PaO_2_:FiO_2_ ratio < 40 kPa (300 mmHg) or arterial oxyhemoglobin saturation measured with pulse oximetry < 90% and requiring oxygen therapy
Pleural effusion	Chest radiograph demonstrating blunting of the costo-phrenic angle, loss of sharp silhouette of the ipsilateral hemidiaphragm in upright position, evidence of displacement of adjacent anatomical structures or (in supine position) a hazy opacity in one hemithorax with preserved vascular shadows
Atelectasis	Lung opacification with a shift of the mediastinum, hilum or hemidiaphragm toward the affected area, and compensatory over-inflation in the adjacent non-atelectatic lung
Pneumothorax	Air in the pleural space with no vascular bed surrounding the visceral pleura
Aspiration pneumonitis	Acute lung injury after the inhalation of regurgitated gastric contents

**Table 2:** Patient characteristics for unmatched cohort and propensity score-matched cohort.

Variables	Unmatched Cohort (*n* = 3261)			Matched Cohort (*n* = 1232)			
	Sugammadex	Neostigmine	*p*-Value	Sugammadex	Neostigmine	*p*-Value	SMD
	(*n* = 1363)	(*n* = 1898)		(*n* = 616)	(*n* = 616)		
Patient-related							
Age (year)	60.5 (12.8)	59.9 (12.4)	0.208	63.5 (11.7)	62.9 (11.6)	0.328	0.016
Sex: Male	859 (63%)	1192 (62.8%)	0.898	423 (68.7%)	424 (68.8%)	0.951	0.004
Height (cm)	163 (8.7)	163.1 (9.1)	0.711	163.4 (8.8)	163.3 (8.7)	0.732	0.018
Weight (kg)	63.6 (11.6)	64.1 (11.3)	0.209	64.7 (11)	64.3 (11.3)	0.72	0.02
Body mass index (kg/m^2^)	23.9 (3.4)	24 (3.2)	0.198	23.9 (3.3)	24 (3.3)	0.523	0.071
ASA classification			0.341			0.863	0.031
1	600 (44%)	841 (44.3%)		231 (37.5%)	235 (38.1%)		
2	700 (51.4%)	1645 (53.2%)		363 (58.9%)	356 (57.8%)		
3	62 (4.5%)	34 (1.1%)		22 (3.6%)	25 (4.1%)		
4	1 (0.1%)	0		0	0		
Anemia	62 (4.5%)	96 (5.1%)		25 (4.1%)	25 (4.1%)	1	<0.001
GFR (mL/min/1.73 m^2^)			0.683			1	<0.001
GFR ≥ 60	1297 (95.2%)	1807 (95.3%)		585 (95%)	585 (95%)		
30 ≤ GFR < 60	63 (4.6%)	88 (4.6%)		31 (5%)	31 (5%)		
GFR < 30	2 (0.1%)	1 (0.1%)		0	0		
Hypertension	467 (34.3%)	663 (34.9%)	0.692	240 (39%)	238 (38.6%)	0.907	0.007
Diabetes Mellitus	234 (17.2%)	310 (16.3%)	0.528	118 (19.2%)	124 (20.1%)	0.667	0.025
Heart disease	91 (6.7%)	104 (5.5%)	0.155	41 (6.7%)	46 (7.5%)	0.578	0.032
Brain disease	55 (4%)	72 (3.8%)	0.725	26 (4.2%)	23 (3.7%)	0.662	0.025
Smoking history			0.732			0.954	0.017
Never smoker	704 (52%)	1009 (53.4%)		296 (48.1%)	300 (48.7%)		
Ex-smoker	407 (30.1%)	555 (29.4%)		200 (32.5%)	195 (31.7%)		
Current smoker	242 (17.9%)	325 (17.2%)		120 (19.5%)	121 (19.6%)		
Preoperative lung disease			0.094			0.947	0.085
None	1262 (92.6%)	1798 (94.7%)		562 (91.2%)	566 (91.9%)		
Asthma	13 (1.4%)	18.6 (1%)		7 (1.1%)	6 (1%)		
COPD	32 (2.3%)	38.4 (1.8%)		16 (2.6%)	19 (3.1%)		
Old Tb	17 (1.2%)	23 (1.2%)		9 (1.5%)	9 (1.5%)		
Tb destroyed lung	4 (0.3%)	3 (0.2%)		3 (0.5%)	3 (0.5%)		
Lung cancer	11 (0.8%)	7 (0.4%)		7 (1.1%)	3 (0.5%)		
Others	16 (1.2%)	9 (0.5%)		9 (1.5%)	8 (1.3%)		
Combination	8 (0.6%)	5 (0.3%)		3 (0.5%)	2 (0.3%)		
Pulmonary Function Test			0.071			0.994	0.016
FEV1/FVC ≥ 70%	787 (76.3%)	980 (73%)		461 (74.8%)	457 (74.2%)		
FEV1 ≥ 80%, FVC < 70%	181 (17.5%)	288 (21.4%)		121 (19.6%)	125 (20.3%)		
50 ≤ FEV1 < 80, FVC < 70%	54 (5.2%)	68 (5.1%)		28 (4.5%)	28 (4.5%)		
30 ≤ FEV1 < 50, FVC < 70%	10 (1%)	7 (0.5%)		6 (1%)	6 (1%)		
Cancer and Surgery-related							
Type of operation			0.000			0.791	0.088
Gastric wedge resection	92 (6.7%)	122 (6.4%)		36 (5.8%)	40 (6.5%)		
LADG	818 (60%)	1307 (68.9%)		396 (64.3%)	381 (61.9%)		
LAPG	164 (12%)	141 (7.4%)		54 (8.8%)	69 (11.2%)		
LATG	139 (10.2%)	201 (10.6%)		64 (10.4%)	62 (10.1%)		
Pylorus preserving gastrectomy	27 (2%)	27 (1.4%)		15 (2.4%)	15 (2.4%)		
TLDG	123 (9%)	100 (5.3%)		51 (8.3%)	49 (8%)		
Diagnosis			0.052			0.545	0.1
EGC	834 (61.2%)	1167 (61.5%)		368 (59.7%)	373 (60.6%)		
AGC	422 (31%)	582 (30.7%)		206 (33.4%)	193 (31.3%)		
Benign	18 (1.3%)	20 (1.1%)		3 (0.5%)	6 (1%)		
NEC	15 (1.1%)	6 (0.3%)		3 (0.5%)	1 (0.2%)		
GIST	74 (5.4%)	122 (6.4%)		36 (5.8%)	43 (7%)		
Anesthesia-related							
Anesthetic agent			0.000			0.293	0.089
Total Intravenous Anesthesia	120 (9%)	316 (16.9%)		64 (10.4%)	66 (10.7%)		
Desflurane	1118 (83.6%)	972 (51.9%)		480 (77.9%)	460 (74.7%)		
Sevoflurane	99 (7.4%)	584 (31.2%)		72 (11.7%)	90 (14.6%)		
Anesthetic time (min)	220 (68.4)	226 (70)	0.46	222.2 (70.3)	221.4 (63.5)	0.837	0.012
Positive End Expiratory Pressure	774 (56.8%)	497 (26.2%)	0.000	261 (42.4%)	250 (40.6%)	0.525	0.036
Peak Inspiratory Pressure (mmHg)	18 (3.6)	18 (3.5)	0.168	18 (3.6)	18 (3.3)	0.658	0.025
Crystalloid (cc)	1085.4 (464.4)	1118 (492.5)	0.057	1101.4 (484.7)	1093.7 (454.4)	0.775	0.016
Colloid (cc)	31.7 (127.7)	51.3 (167.2)	0.000	43.4 (153.1)	33.7 (135)	0.241	0.07
Estimated Blood Loss (cc)	50.8 (104.3)	75.2 (119)	0.000	57.6 (133.7)	56.3 (83.8)	0.833	0.012
Urine Output (cc)	133.6 (130)	145.5 (160.1)	0.024	139.6 (138.8)	138.3 (130.3)	0.866	0.01
Transfusion (cc)	0.8 (15)	1.4 (20)	0.306	0.97 (13.9)	0.73 (12.9)	0.318	0.018
Phenylephrine continuous infusion	48 (3.5%)	62 (3.3%)	0.691	21 (3.4%)	27 (4.4%)	0.377	0.05
Norepinephrine continuous infusion	17 (1.2%)	8 (0.4%)	0.008	9 (1.5%)	4 (0.6%)	0.163	0.08
Dopamine continuous infusion	6 (0.4%)	5 (0.3%)	0.391	4 (0.6%)	2 (0.3%)	0.413	0.047
Dobutamine continuous infusion	1 (0.1%)	0 (0%)	0.238	0	0		<0.001
Nitroglycerin continuous infusion	6 (0.4%)	9 (0.5%)	0.888	3 (0.5%)	1 (0.2%)	0.317	0.057
Ephedrine	936 (68.7%)	1127 (59.4%)	0.000	406 (65.9%)	409 (66.4%)	0.857	0.01
Phenylephrine	587 (43.1%)	559 (29.5%)	0.000	236 (38.3%)	229 (37.2%)	0.681	0.023
Atropine	34 (2.5%)	80 (4.2%)	0.008	20 (3.2%)	12 (1.9%)	0.152	0.082
Esmolol	81 (5.9%)	185 (9.7%)	0.000	46 (7.5%)	44 (7.1%)	0.827	0.012

Presented as number (%) or mean (standard deviation). SMD, standardized mean difference; ASA, American Society of Anesthesiologists; GFR, Glomerular Filtration Rate; COPD, Chronic Obstructive Pulmonary Disease; Tb, tuberculosis; FEV1, Forced Expiratory Volume in 1 s; FVC, forced vital capacity; LADG, Laparoscopic Assisted Distal Gastrectomy; LAPG, Laparoscopic Assisted Proximal Gastrectomy; LATG, Laparoscopic Assisted Total Gastrectomy; TLDG, Totally Laparoscopic distal gastrectomy; EGC, Early Gastric Cancer; AGC, Advanced Gastric Cancer; NEC, Neuroendocrine Carcinoma; GIST, Gastrointestinal Stromal Tumor.

**Table 3:** Postoperative pulmonary complication rate in the propensity-matched cohort.

	Sugammadex (*n* = 616)	Neostigmine (*n* = 616)	*p* Value
Total	286 (46.4%)	304 (49.4%)	0.305
Respiratory infection	12 (1.9%)	6 (1.0%)	0.154
Respiratory failure	3 (0.5%)	3 (0.5%)	1
Pleural effusion	111 (18.0%)	144 (23.4%)	0.02 ^1^
Atelectasis	223 (36.2%)	219 (35.6%)	0.812
Pneumothorax	3 (0.5%)	4 (0.6%)	0.705
Aspiration pneumonitis	0 (0.0%)	1 (0.2%)	0.317
Others	1 (0.2%)	3 (0.5%)	0.317

Presented as number (%). ^1^ *p* < 0.05.

**Table 4:** Secondary outcomes in the propensity-matched cohort.

	Sugammadex (*n* = 616)	Neostigmine (*n* = 616)	*p* Value
Re-operation within 90 days	17 (2.1%)	13 (2.1%)	1
Postoperative ICU admission	44 (7.1%)	48 (7.8%)	0.665
Re-admission or emergency room visit within 30 days	58 (9.4%)	69 (11.2%)	0.303
Length of hospital stay	8.72 (4.1)	9.09 (6.6)	0.238
Death within 90days	1 (0.2%)	0 (0.0%)	0.317

Presented as number (%) or mean (standard deviation). ICU, Intensive Care Unit.
